# Total synthesis of the indolizidine alkaloid tashiromine

**DOI:** 10.1186/1860-5397-4-8

**Published:** 2008-01-26

**Authors:** Stephen P Marsden, Alison D McElhinney

**Affiliations:** 1 School of Chemistry, University of Leeds, Leeds LS2 9JT, UK

## Abstract

**Background:**

Tashiromine (**1**) is a naturally occurring indolizidine alkaloid. It has been the subject of thirteen successful total syntheses to date. Our own approach centres on the stereoselective construction of the indolizidine core by capture of an electrophilic acyliminium species by a pendant allylsilane. The key cyclisation precursor is constructed using olefin cross-metathesis chemistry, which has the potential to facilitate both racemic and asymmetric approaches, depending upon the choice of the allylsilane metathesis partner.

**Results:**

The use of the allyltrimethylsilane cross-metathesis approach enables the rapid construction of the key cyclisation precursor **3** (3 steps from commercial materials), which undergoes acid-induced cyclisation to give the desired bicyclic indolizidine skeleton as a 96:4 mixture of diastereomers. Simple functional group interconversions allowed the completion of the total synthesis of racemic tashiromine in six steps (19% overall yield). Three chiral α-alkoxyallylsilanes (**12**,** 14** and **15**) were prepared in enantioenriched form and their cross-metathesis reactions studied as part of a putative asymmetric approach to tashiromine. In the event, α-hydroxysilane **12** underwent isomerisation under the reaction conditions to acylsilane **17**, while silanes **14** and **15** were unreactive towards metathesis.

**Conclusion:**

A concise, stereoselective total synthesis of racemic tashiromine has been developed. Attempts to translate this into an asymmetric synthesis have thus far been unsuccessful.

## Background

**Scheme 1:**
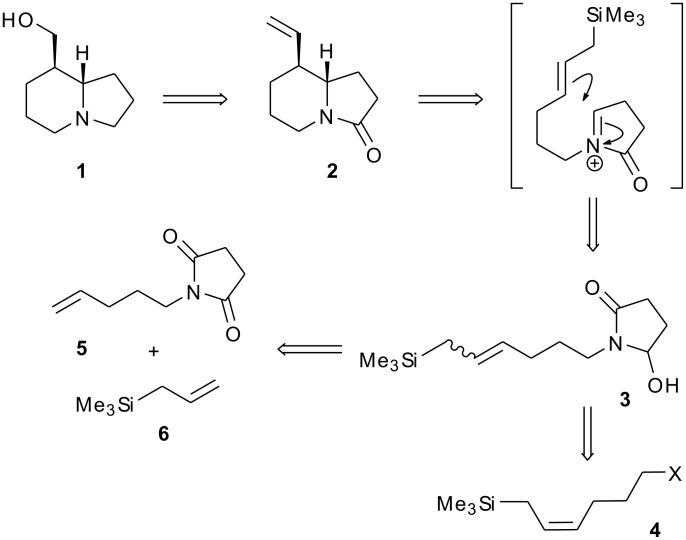
Retrosynthesis for tashiromine.

Tashiromine (**1**) is a naturally occurring indolizidine, isolated from an Asian deciduous shrub *Maackia tashiroi* [1]. As one of the structurally simpler indolizidine alkaloids [2], tashiromine has been a popular target for synthetic chemists, and to date has succumbed to total synthesis on thirteen occasions [3–15]. A wide variety of reactions have been employed to assemble the core indolizidine structure, including radical cyclisations [3]; nucleophilic addition to imines [5,14–15]; electrophilic alkylation of pyrroles [7,13]; alkylation of enamines [6], β-amino esters [8] and pyrrolidinyllithiums [12]; stereoselective reduction of enamines [4,9] and pyridinium salts [11]; and titanium-mediated reductive imide-olefin cyclisation [10]. Our own approach [14] utilises an intramolecular addition of an allylsilane to an *N*-acyliminium ion to deliver the [4.3.0]-azabicyclic (indolizidine) skeleton **2** (Scheme 1), wherein the pendant vinyl group acts as a handle to install the hydroxymethyl sidechain found in tashiromine. The synthesis of azabicyclic assemblies by intramolecular allylsilane/*N*-acyliminium cyclisations was first studied by Hiemstra and Speckamp [16], who prepared their functionalised allylsilane cyclisation precursors (such as **3**) by alkylation of cyclic imides with reagent **4** (X = OMs). This, in turn, was prepared in four steps by alkylation of an acetylide anion with commercially available iodomethyltrimethylsilane, followed by partial reduction of the alkyne. Alternative synthetic approaches to **4** (X = OMs, I) involve olefination of aldehydes using the Seyferth-Fleming phosphorane [17] or nickel-catalysed 1,2-metallate rearrangement of lithiated dihydropyran [18]. Our approach was informed by the prior work by our own group [19–24] and others [25–38] on the use of olefin metathesis to generate functionalised allylsilanes. Specifically, cross-metathesis of *N*-pentenylsuccinimide **5** with allyltrimethylsilane (**6**) [39] followed by chemoselective partial reduction of the imide would give the cyclisation precursor **3** in short order. Further, the use of chiral allylsilanes as cross-metathesis partners would potentially facilitate an asymmetric approach to the total synthesis of **1**. We report herein full details of the successful synthesis of racemic tashiromine **1** by this strategy [14], as well as our initial attempts towards an asymmetric variant.

## Results and Discussion

**Scheme 2:**
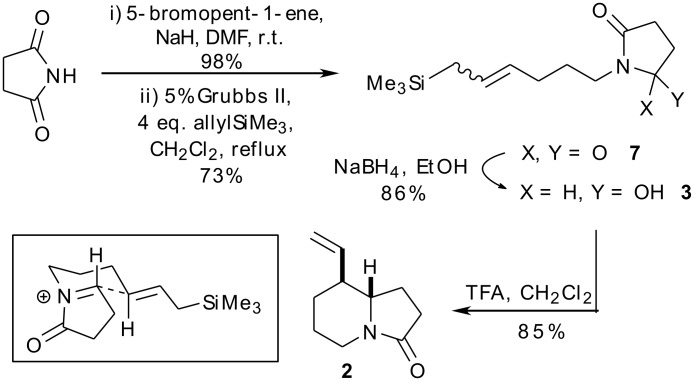
Stereoselective construction of the indolizidine core **2**.

Metathesis precursor **5** was prepared by alkylation of the sodium salt of succinimide with 5-bromo-1-pentene in near quantitative yield (Scheme 2, see Supporting Information File 1 for full experimental data). The key cross-metathesis reaction of **5** was carried out using a fourfold excess of allyltrimethylsilane (**6**) and 5 mol% of Grubbs’ second generation catalyst in refluxing dichloromethane. The desired product **7** was formed in 73% yield as an inseparable 3:1 mixture of *E-* and *Z*-isomers. Partial reduction with sodium borohydride generated the cyclisation precursor **3** in 86% yield, again as a 3:1 mixture of olefin isomers. Exposure of this mixture to trifluoroacetic acid in dichloromethane at room temperature gave the bicyclic amide **2** in 85% yield as a 96:4 mixture of diastereomers. The identity of the major diastereomer was confirmed by comparison of the spectral data with those of Hiemstra [16]: specifically, the signal for the (ring-fusion) proton at C6 for the major diastereomer appeared as a doublet of triplets with δ = 3.19 ppm, whereas the corresponding signal for the minor diastereomer appeared at δ = 3.67 ppm. The stereochemical outcome of this reaction was rationalised on the basis of the model shown in Scheme 2, whereby nucleophilic addition of the allylsilane to the *N*-acyliminium ion occurs through a chair-like transition state with the nascent alkene equatorially disposed.

**Scheme 3:**
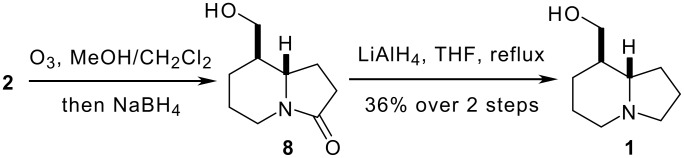
Completion of the total synthesis of tashiromine** 1**.

All that remained to complete the synthesis of tashiromine **1** was to effect the oxidative cleavage of the C5 vinyl substituent, then carry out a global reduction of the resulting carbonyl function and the amide. In the event, attempts to form a C5 aldehyde using either ozonolytic or dihydroxylation/periodate alkene cleavage protocols were unsuccessful, with complex mixtures being obtained in both cases. We suspected that the problem lay in the potential for the desired aldehyde to undergo retro-Mannich fragmentation, and so elected to carry out a reductive work-up to the ozonolysis procedure (Scheme 3). The desired alcohol **8** was obtained in a crude form and immediately subjected to reduction with lithium aluminium hydride to give our target tashiromine **1** in 36% yield over two steps. Our stereochemical assignment for the cyclisation of **3** was further corroborated by the agreement of the spectral data for **1** with those previously published in the literature [3–5,9–12]. Additionally, the spectral data for the diastereomeric *epi*-tashiromine have been reported and differ significantly from those recorded for **1** [10].

**Figure 1:**
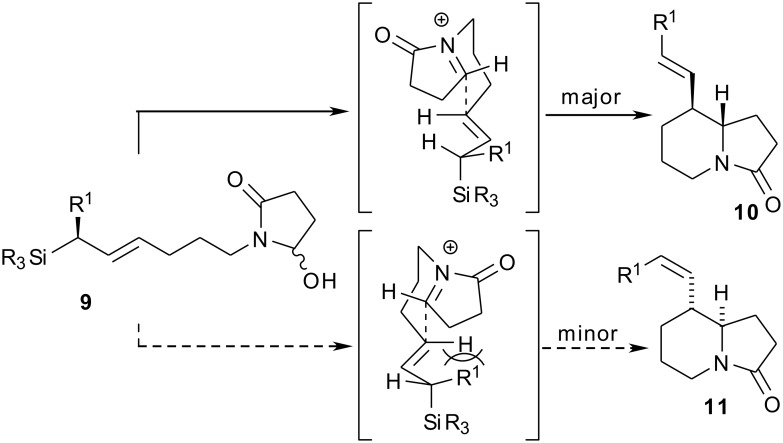
Rationale for stereoselective assembly of the indolizidine core using chiral allylsilanes.

Having completed our target synthesis, our next goal was to investigate an asymmetric approach to tashiromine. Specifically, we envisaged that cyclisation precursors of type **9** ought to be readily available by cross-metathesis of **5** with an appropriate chiral allylsilane followed by chemoselective partial reduction by borohydride. Thereafter, exposure to acid would generate an *N*-acyliminium ion, which would cyclise through a chair-like transition state with the nascent alkenyl side-chain equatorially disposed, as in the racemic series (Figure 1). The absolute stereochemistry of the newly established asymmetric centres would be controlled by allylic strain arguments, assuming that the well-established precedent for *anti*-S_E_2’ attack of the iminium on the allylsilane was upheld here [40]. Thus, the predicted major stereoisomer **10** would have (5*S*,6*S*) stereochemistry and an *E*-configured side-chain, while cyclisation to the predicted minor (5*R*,6*R*) isomer **11** would be disfavoured by A_1,3_-interactions between the R^1^ group and vinylic proton (leading to the *Z*-configured side-chain). This would represent an immolative transfer of chirality approach to tashiromine, since the olefinic side-chains would be cleaved to install the hydroxymethyl side-chain required by the natural product.

**Scheme 4:**
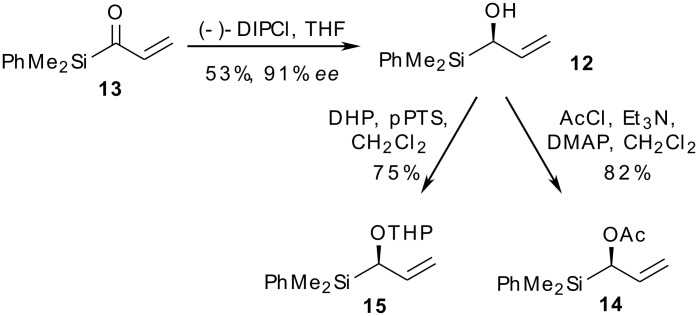
Asymmetric synthesis of chiral (alkoxy)allylsilanes.

Our approach centred on the readily availability of chiral α-hydroxysilane **12** in enantioenriched format [41]. Protection of the hydroxyl group, either before or after cross-metathesis, would allow access to chiral allylsilanes **9** with R^1^ being an alkoxy or acyloxy group. Furthermore, this would generate products **10** and/or **11** with a readily oxidised enol-ether/ester side chain for progression to tashiromine. We were, of course, mindful that these functions could potentially act as nucleophiles themselves in the acidic medium of the electrophilic cyclisation, and the investigation of such chemoselectivity issues provided a further impetus for this study. Acylsilane **13** was therefore prepared from propargyl alcohol in four steps then subjected to asymmetric reduction with (−)-DIPCl according to Buynak *et al.* (Scheme 4) [41]. The desired hydroxysilane **12** was obtained in 53% yield and with 91% *ee* as determined by chiral HPLC analysis. Compound **12** was converted by standard methods to the acetate **14** and the tetrahydropyranyl ether **15**. The latter compound was formed as a 1.3:1 mixture of diastereomers which were partially separated by column chromatography — all subsequent reactions were carried out on diastereomerically pure material for ease of analysis.

**Scheme 5:**
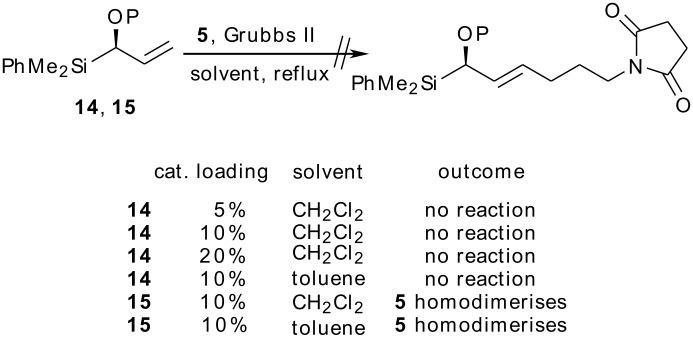
Attempted cross-metathesis of (alkoxy)allylsilanes.

With the requisite enantioenriched allylsilanes in hand, we next investigated their behaviour in olefin cross-metathesis reactions. Unfortunately, neither **14** nor **15** reacted with **5** under the standard cross-metathesis conditions used for trimethylsilane **6**; the use of more forcing conditions (elevated temperature and higher catalyst loadings) did not effect the desired transformation, the only product observed being that of homodimerisation of **5** (Scheme 5).

**Scheme 6:**
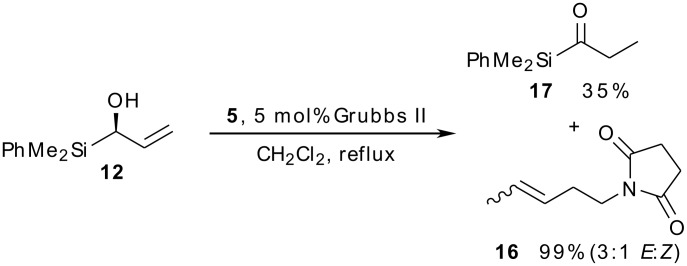
Competing isomerisation processes in attempted cross-metathesis of (hydroxy)allylsilane **12**.

Finally, we examined the behaviour of alcohol **12** under cross-metathesis conditions. In the event, two isomerised products were isolated from this reaction (Scheme 6): the internal alkene **16** (formed in 99% yield as a ca. 3:1 mixture of *E:Z* isomers) and the acylsilane **17**. The formation of isomerised alkenes accompanying (or instead of) metathesis processes using ruthenium-based catalysts is well documented [42–63], as is the formation of carbonyl compounds by isomerisation of the corresponding allylic alcohols [64–68]. At this stage we therefore reluctantly abandoned our investigations into the asymmetric synthesis of tashiromine.

## Conclusion

A concise, stereoselective total synthesis of racemic tashiromine has been developed (six steps from succinimide, 19% overall yield) in which the key steps are the preparation of a functionalised allylsilane by olefin cross-metathesis and the construction of the indolizidine core by intramolecular addition of the allylsilane to an *N*-acyliminium ion. Attempts to translate this into an asymmetric synthesis utilising cross-metathesis reactions of chiral α-alkoxysilanes have thus far been unsuccessful.

## Experimental

Experimental protocols for the synthesis of tashiromine **1** and the preparation of silanes **12**, **14**, **15** and **17** available as Supporting Information File 1.

## Supporting Information

File 1 Supporting Information. Full experimental details and compound characterisation data for all new compounds described.
