# Supplementary material for: Total synthesis of the indolizidine alkaloid tashiromine
Source: Beilstein J Org Chem. 2008 Jan 26;4:8. doi: 10.1186/1860-5397-4-8 (PMC2241604; doi:10.1186/1860-5397-4-8)
Supplement: File 1 — Supporting Information. Full experimental details and compound characterisation data for all new compounds described. [file Beilstein_J_Org_Chem-04-08-s001.pdf]

Total synthesis of the indolizidine alkaloid tashiromine

Stephen P. Marsden* and Alison D. McElhinney

School of Chemistry, University of Leeds, Leeds LS2 9JT, UK

s.p.marsden@leeds.ac.uk

Supporting Information

1-Pent-4-enylpyrrolidine-2,5-dione 5

Succinimide (0.66 g, 6.7 mmol) was dissolved in anhydrous DMF (3.3 mL) and was cooled to 0 ^{o}C. Sodium hydride (0.29 g of a 60% dispersion in mineral oil, 7.4 mmol) was added and the mixture stirred for 5 min at 0 ^{o}C then for 1 h at room temperature. 5-Bromopent-1-ene (0.79 mL, 6.7 mmol) was added dropwise and the reaction stirred for a further 3 h at room temperature. The reaction mixture was CAUTIOUSLY poured into a mixture of water (10 mL) and a saturated aqueous solution of ammonium chloride (10 mL). The product was extracted with Et_2O (3 × 15 mL) and the combined organic phases washed with water (10 mL), dried over $MgSO_4$ and the solvent removed *in vacuo*. Purification by column chromatography (hexane:EtOAc, 6:4) yielded *1-pent-4-enylpyrrolidine-2,5-dione* **5** (1.10 g, 98%) as a colourless oil, whose data is ν_{max}/cm^{-1} (Liquid film) 2942 and 2866 (w, aliphatics), 1773 and 1698 (CO-NH-CO), 1641 (C=C); δ_H (300 MHz, $CDCl_3$) 5.80 (1H, ddt, *J* 16.9, 10.2, 6.9, C**H**=CH_2), 5.04 (1H, dd, *J* 16.9, 1.5, CH=C(**H**)H *trans*), 4.99 (1H, d, *J* 10.2, CH=C(**H**)H *cis*), 3.52 (2H, t, *J* 7.7, NC$\mathbf{H_2}$), 2.71 (4H, s, COC$\mathbf{H_2}$C$\mathbf{H_2}$CO), 2.07 (2H, q, *J* 7.2, C$\mathbf{H_2}$C=C), 1.68 (2H, quintet, *J* 7.7, CH_2C$\mathbf{H_2}$$CH_2$); δ_C (75 MHz, $CDCl_3$) 177.7 (**C**=O), 137.7 (**C**H=CH_2), 115.7 (CH=**C**H_2), 38.8 (N**C**H_2), 31.4 (**C**H_2C=C), 28.5 (CO**C**H_2), 27.1 (CH_2**C**$H_2$$CH_2$). LRMS m/z (ES) 316.9 (51%), 220.8 (64), 167.7 (53) $[M+H]^+$. Data corresponds to that quoted in the literature. [1]

***1-(6-Trimethylsilylhex-4-enyl)pyrrolidin-2,5-dione* 7**

1-Pent-4-enylpyrrolidine-2,5-dione **5** (0.74 g, 4.4 mmol) was dissolved in anhydrous degassed CH_2Cl_2 (70 mL, 0.063 M) and was heated to reflux for 30 min. Allyltrimethylsilane **6** (2.8 mL, 17.7 mmol) was added *via* a syringe followed by the addition of the second generation Grubbs' catalyst tricyclohexylphosphine[1,3-bis(2,4,6-trimethylphenyl)-4,5-dihydroimidazol-2-ylidene][benzylidene]ruthenium (IV) dichloride (0.19 g, 0.22 mmol, 5 mol%). The solution became a rose colour and was heated to reflux for 20 h by which time the colour had changed to a golden brown. The reaction vessel was opened to air and stirred for 3 h. The volume of solvent was reduced *in vacuo* to 2 mL and this solution flushed through a plug of silica gel with CH_2Cl_2 (50 mL) followed by 16% EtOAc in hexanes (100 mL). The solvents were removed *in vacuo* and the residue purified by column chromatography (petrol:EtOAc, 6:4) to yield *1-(6-trimethylsilylhex-4-enyl)pyrrolidin-2,5-dione* **7** (0.82 g, 73%) as a colourless oil. An inseparable mixture of stereoisomers was obtained (*Z:E* 1:3), whose data is ν_{max}/cm^{-1} (Liquid film) 2952 and 2899 (m, aliphatics), 1775 and 1701 (CO-NH-CO). Data for the major *E*-stereoisomer: δ_H (300 MHz, $CDCl_3$) 5.45 (1H, dt, *J* 14.8, 7.2, CH=C**H**CH$_2$Si), 5.22 (1H, dt, *J* 14.8, 7.2, C**H**=CHCH$_2$Si), 3.51 (2H, t, *J* 7.7, NC**H$_2$**), 2.71 (4H, s, COC**H$_2$**C**H$_2$**CO), 2.01 (2H, q, *J* 7.2, C**H$_2$**C=C), 1.62 (2H, quintet, *J* 7.7, CH$_2$C**H$_2$**CH$_2$), 1.42 (2H, d, *J* 7.2, SiC**H$_2$**), 0.00 (9H, s, Si(C**H$_3$**)$_3$); δ_C (75 MHz, $CDCl_3$) 179.3 (**C**=O), 129.3 (**C**H=CHCH$_2$Si), 129.0 (CH=**C**HCH$_2$Si), 40.5 (N**C**H$_2$), 32.1 (**C**H$_2$C=C), 30.1 (CO**C**H$_2$), 29.8 (CH$_2$**C**H$_2$CH$_2$), 24.7 (**C**H$_2$Si), 0.0 (Si(**C**H$_3$)$_3$). Signals for the minor *Z*-stereoisomer visible at: δ_H (300 MHz, $CDCl_3$) 3.56 (2H, t, *J* 7.2, NC**H$_2$**), 2.70 (4H, s, COC**H$_2$**C**H$_2$**CO), 1.47 (2H, d, *J* 8.7, SiC**H$_2$**), 0.01 (9H, s, Si(C**H$_3$**)$_3$); δ_C (75 MHz, $CDCl_3$) 128.6 (**C**H=CHCH$_2$Si), 127.7 (CH=**C**HCH$_2$Si), 40.6 (N**C**H$_2$), 29.6 (**C**H$_2$C=C), 26.4 (CO**C**H$_2$), 24.7 (CH$_2$**C**H$_2$CH$_2$), 20.5 (**C**H$_2$Si), 0.2 (Si(**C**H$_3$)$_3$). LRMS m/z (ES) 253.9 (12%) $[M+H]^+$, 241.8 (33), 209.8 (100).

Data for the *Z*-isomer corresponds to that quoted in the literature. [2]

***5-Hydroxy-1-(6-trimethylsilylhex-4-enyl)pyrrolidin-2-one* 3**

1-(6-Trimethylsilylhex-4-enyl)pyrrolidin-2,5-dione **7** (0.50 g, 2.0 mmol) was dissolved in ethanol (12 mL) and cooled to 0 ^{o}C. Sodium borohydride (0.37 g, 9.9 mmol) was added in one portion. A

solution of 2 N HCl (in a 4:1 mixture of water:ethanol, 4-6 drops) was added every 15 min maintaining the temperature at 0 °C. After 4 h the reaction mixture was diluted with Et_2O (30 mL) and poured into cold water (20 mL). The organic layer was isolated and the aqueous phase extracted with further portions of Et_2O (2 × 20 mL). The combined organic phases were washed with water (20 mL), brine (20 mL), dried over $MgSO_4$ and the solvent removed *in vacuo*. Purification by column chromatography (EtOAc) yielded *5-hydroxy-1-(6-trimethylsilylhex-4-enyl)pyrrolidin-2-one* **3** (0.44 g, 86%) as a colourless oil. An inseparable mixture of stereoisomers was obtained (*Z*:*E*, 1:3), whose data is ν_{max}/cm^{-1} (Liquid film) 3338 (s, OH), 2952 (m, aliphatics), 1667 (NHCO). Data for the major *E*-stereoisomer: δ_H (300 MHz, $CDCl_3$) 5.42 (1H, dt, *J* 15.3, 7.7, CH=C**H**CH_2Si), 5.30-5.19 (3H, m, C**H**=CHCH_2Si and C**H**O**H**), 3.53-3.43 (1H, m, NC(**H**)H), 3.21-3.10 (1H, m, NC(**H**)H), 2.61-2.47 (1H, m, COC(**H**)H), 2.35-2.20 (2H, m, COC(**H**)H and C(**H**)HCHOH), 2.05-1.88 (3H, m, C(**H**)HCHOH and C$\mathbf{H_2}$C=C), 1.70-1.53 (2H, m, CH_2C$\mathbf{H_2}$$CH_2$), 1.41 (2H, d, *J* 7.7, SiC$\mathbf{H_2}$), 0.00 (9H, s, Si(C$\mathbf{H_3}$)$_3$); δ_C (75 MHz, $CDCl_3$) 177.1 (**C**=O), 129.4 (**C**H=CHCH_2Si), 128.9 (CH=**C**HCH_2Si), 85.0 (**C**HOH), 41.6 (N**C**H_2), 32.2 (**C**H_2C=C), 31.0 (CO**C**H_2), 30.1 (**C**H_2CHOH), 29.8 (CH_2**C**$H_2$$CH_2$), 24.6 (**C**$H_2$Si), 0.0 (Si(**C**$H_3$)$_3$). Signals for the minor *Z*-stereoisomer visible at: δ_H (300 MHz, $CDCl_3$) 1.46 (2H, d, *J* 8.7, SiC$\mathbf{H_2}$), 0.02 (9H, s, Si(C$\mathbf{H_3}$)$_3$); δ_C (75 MHz, $CDCl_3$) 128.3 (**C**H=CHCH_2Si), 128.1 (CH=**C**HCH_2Si), 41.6 (N**C**H_2), 29.6 (**C**H_2C=C), 26.5 (CH_2**C**$H_2$$CH_2$), 20.5 (**C**$H_2$Si), 0.1 (Si(**C**$H_3$)$_3$).

LRMS m/z (ES) 339.9 (52%), 255.9 (46) $[M+H]^+$, 231.8 (47), 224.9 (100).

Data corresponds to that for the minor *Z*-stereoisomer quoted in the literature. [2]

***(±)-(5RS, 6RS)-5-Vinyl-1-azabicyclo[4.3.0]nonan-9-one* 2**

A solution of TFA (0.51 mL) in CH_2Cl_2 (25 mL) was added dropwise *via* a cannula to a solution of 5-hydroxy-1-(6-trimethylsilylhex-4-enyl)pyrrolidin-2-one **3** (0.42 g, 1.6 mmol) in CH_2Cl_2 (5.9 mL) cooled to 0 °C. The reaction was for 1 h stirred at room temperature then diluted with CH_2Cl_2 (20 mL). The organic phase was washed with a saturated aqueous solution of sodium bicarbonate (20 mL) and the basic solution re-extracted with CH_2Cl_2 (2 × 10 mL). The combined organic phases were washed with water (20 mL), brine (20 mL), dried over $MgSO_4$ and the solvent

removed *in vacuo*. Purification by column chromatography (EtOAc) yielded *(±)-5-vinyl-1-azabicyclo[4.3.0]nonan-9-one* **2** (0.23 g, 85% of an inseparable 96:4 mixture of diastereomers) as a colourless oil, whose data is ν_{max}/cm^{-1} (Liquid film) 2934 and 2856 (w, aliphatics), 1686 (NHCO). Data for the major diastereomer: δ_H (300 MHz, $CDCl_3$) 5.68 (1H, ddd, *J* 17.9, 10.2, 7.7, C**H**=CH_2), 5.16 (1H, dd, *J* 17.9, 1.0, CH=C(**H**)H *trans*), 5.12 (1H, dd, *J* 10.2, 1.0 CH=C(**H**)H *cis*), 4.19-4.12 (1H, m, 1 × **H2**), 3.19 (1H, dt, *J* 9.7, 7.2, **H6**), 2.63 (1H, td, *J* 12.2, 2.6, 1 × **H2**), 2.43-1.27 (9H, m, **H3**-**H5**, **H7** and **H8**); δ_C (75 MHz, $CDCl_3$) 172.7 (**C**=O), 137.4 (**C**H=CH_2), 115.2 (CH=**C**H_2), 59.7 (**C6**), 47.6 (**C2**), 38.8 (**C5**), 29.2 (**C8**), 29.0 (**C4**), 23.0 (**C3**), 22.6 (**C7**).
Signals for the minor diastereomer visible at: δ_H (300 MHz, $CDCl_3$) 5.93 (1H, m, C**H**=CH_2), 3.67 (1H, m, **H6**); δ_C (75 MHz, $CDCl_3$) 134.3 (**C**H=CH_2), 117.3 (CH=**C**H_2), 42.0 (**C5**), 19.9 (**C3**), 18.0 (**C7**). LRMS m/z (ES) 331.2 (100%), 317.2 (31), 207.1 (29), 166.1 (51) $[M+H]^+$.
Data corresponds to that quoted in the literature. [2]

(±)-(5SR, 6RS)-1-Aza-5-(hydroxymethyl)bicyclo[4.3.0]nonane ((±)-Tashiromine) **1**

Azabicycle **2** (0.15 g, 0.91 mmol) was dissolved in a mixture of CH_2Cl_2 (3 mL) and methanol (3 mL) and was cooled to -78 °C. The reaction mixture was exposed to ozone for 15 min until a light blue colouration noted. N_2 was then bubbled through the solution to remove any excess ozone until the blue colouration was dispersed. Sodium borohydride (69 mg, 1.82 mmol) was added and the reaction allowed to warm to room temperature. The solution was concentrated and the residue dissolved in CH_2Cl_2 (5 mL) and water added carefully (1 mL). The organic layer was isolated and the aqueous phase re-extracted with further portions of CH_2Cl_2 (3 × 2 mL). The combined organic phases were dried over $MgSO_4$ and the solvent removed *in vacuo*. The crude alcohol (90 mg) was dissolved in THF (6 mL) and added dropwise *via* a cannula to a suspension of lithium aluminium hydride (42 mg, 1.12 mmol) in THF (9 mL) at 0 °C. The resultant mixture was heated to reflux for 3 h. The reaction was allowed to cool to room temperature and water (0.2 mL) added carefully. The mixture was filtered through a pad of celite and the salt washed with minimal amounts of water. The filtrate was dried over $MgSO_4$ and the solvent removed *in vacuo*. Purification by column chromatography (Et_2O:methanol:NH_4OH, 85:15:2) yielded *(±)-*

(5SR, 6RS)-1-aza-5-(hydroxymethyl)bicyclo[4.3.0]nonane ((±)-tashiromine) **1** (50 mg, 36% over 2 steps) as a pale yellow oil, whose data is ν_{max}/cm^{-1} (Liquid film) 3356 (OH), 2931 (w, aliphatics); δ_H (300 MHz, $CDCl_3$) 3.68 (1H, dd, *J* 10.8, 4.6, C(**H**)HOH), 3.50 (1H, dd, *J* 10.8, 6.7, C(**H**)HOH), 3.17-3.06 (2H, m, **H2**), 2.11 (1H, q, *J* 9.2, **H6**), 2.04-1.45 (10H, m, **H3**, 1 × **H4**, **H5** and **H7**-**H9**), 1.07 (1H, qd, *J* 11.8, 4.6, 1 × **H4**); δ_C (75 MHz, $CDCl_3$) 66.8 (**C**H_2OH), 66.0 (**H6**), 54.6 (**H2**), 53.1 (**H9**), 45.0 (**H5**), 29.5 (**H4**), 28.0 (**H7**), 25.6 (**H3**), 21.1 (**H8**). No independent signals for the minor diastereomer were visible. LRMS m/z (CI) 156.0 (100%) $[M+H]^+$.

Data corresponds to that quoted in the literature. [3]

(R)-1-(Dimethylphenylsilyl)prop-2-en-1-ol **12**

1-(Dimethylphenylsilyl)propenone **13** [4] (2.8 g, 14.7 mmol) in anhydrous THF (6 mL) was added dropwise *via* a cannula to a solution of (-)-Ipc_2BCl (7.1 g, 22.1 mmol) in anhydrous THF (47 mL). The reaction mixture was stirred for 19 h at room temperature. The solution was concentrated under reduced pressure and the residue dissolved in anhydrous Et_2O (80 mL). Diethanolamine (4.5 mL) was added and the solution stirred at room temperature during which time a white precipitate formed. The precipitate was removed by filtration and was washed with Et_2O. The combined ethereal extracts were washed with 1 N HCl (2 × 75 mL), a saturated aqueous solution of sodium bicarbonate (150 mL), brine (75 mL), dried over $MgSO_4$ and the solvent removed *in vacuo*. Purification by column chromatography (pentane:Et_2O, 9:1) yielded *(R)-1-(dimethylphenylsilyl)prop-2-en-1-ol* **12** (1.5 g, 53%) as a colourless oil, whose data is $[\alpha]_D$ = -6.8; ν_{max}/cm^{-1} (Liquid film) 3412 (OH), 3070 and 3050 (w, aromatics), 2959 and 2902 (w, aliphatics), 1630 (w, C=C); δ_H (300 MHz, $CDCl_3$) 7.58-7.24 (5H, m, **Ph**), 5.99 (1H, ddd, *J* 16.9, 10.7, 5.2, C**H**=CH_2), 5.03 (1H, d, *J* 16.9, CH=C(**H**)H *trans*), 5.00 (1H, d, *J* 10.7, CH=C(**H**)H *cis*), 4.22-4.20 (1H, m, C**H**OH), 0.34 (3H, s, SiC**H₃**), 0.33 (3H, s, SiC**H₃**); δ_C (75 MHz, $CDCl_3$) 139.8 (**C**H=CH_2), 136.5, 134.6, 129.9, 128.3 (**Ph**), 110.4 (CH=**C**H_2), 68.9 (**C**HOH), -5.4 (Si**C**H_3), -5.7 (Si**C**H_3). LRMS m/z (ES) 233.2 (100%), 190.1 (59) $[M-2H]^+$. Data corresponds to that quoted in the literature. [4]

The material was judged to be of 91% *ee* as determined by chiral HPLC. Analytical HPLC: Chiralcel OD-H column. Solvent system: IPA/Hexane, 99% MeCN for 25 min at 1 mL/min. Retention times: **(*S*)-12**, 10.1 min; **(*R*)-12**, 10.8 min.

(R)-Acetic acid 1-(dimethylphenylsilyl)allyl ester 14

(*R*)-1-(Dimethylphenylsilyl)prop-2-en-1-ol **12** (170 mg, 0.88 mmol), triethylamine (140 μl, 0.97 mmol) and dimethylaminopyridine (10 mg) were dissolved in CH_2Cl_2 (4 mL). Acetic anhydride (90 μl, 0.97 mmol) was added and the reaction stirred at room temperature for 19 h. The reaction mixture was washed with water (2 × 10 mL), brine (10 mL), dried over $MgSO_4$ and the solvent removed *in vacuo* to yield *(R)-acetic acid 1-(dimethylphenylsilyl)allyl ester* **14** (170 mg, 82%) as a colourless oil, whose data is $[\alpha]_D$ = +10.1; ν_{max}/cm^{-1} (Liquid film) 2961 (w, aliphatics), 1740 (s, C=O); δ_H (300 MHz, $CDCl_3$) 7.53-7.33 (5H, m, **Ph**), 5.83-5.74 (1H, m, C**H**=CH_2), 5.41-5.37 (1H, m, C**H**OAc), 5.00-4.93 (2H, m, CH=C$\mathbf{H_2}$), 2.04 (3H, s, COC$\mathbf{H_3}$), 0.34 (6H, s, Si(C$\mathbf{H_3}$)$_3$); δ_C (75 MHz, $CDCl_3$) 171.0 (**C**=O), 135.7 (**Ph**), 135.1 (**C**H=CH_2), 134.5, 130.0, 128.3 (**Ph**), 112.2 (CH=**C**H_2), 70.4 (**C**HOAc), 21.4 (CO**C**H_3), -3.4 (Si**C**H_3), -3.7 (Si**C**H_3). LRMS m/z (ES) 235.2 (100%) $[M+H]^+$. Data corresponds to that quoted in the literature. [5]

***(R)-Dimethylphenyl-[1-(tetrahydropyran-2-yloxy)allyl]silane* 15**

(*R*)-1-(Dimethylphenylsilyl)prop-2-en-1-ol **12** (200 mg, 1.0 mmol) was dissolved in CH_2Cl_2 (5 mL). Dihydropyran (140 μl, 1.6 mmol) and *p*PTS (26 mg, 0.10 mmol) were added and the reaction stirred at room temperature for 19 h. The reaction mixture was washed with water (5 mL), brine (5 mL), dried over $MgSO_4$ and the solvent removed *in vacuo*. Purification by column chromatography (pentane:Et_2O, 9.5:0.5) yielded *(R)-dimethylphenyl-[1-(tetrahydropyran-2-yloxy)allyl]silane* **15** as a mixture of THP diastereomers isolated in two fractions.

Combined data: ν_{max}/cm^{-1} (Liquid film) 3070 and 3050 (w, aromatics), 2942, 2868 and 2850 (w, aliphatics), 1627 (w, C=C). **Fraction 1**: An orange oil (92 mg, 33%) was isolated as a single diastereomer. $[\alpha]_D$ = +90.5; Data: δ_H (300 MHz, $CDCl_3$) 7.60-7.24 (5H, m, **Ph**), 5.74-5.63 (1H, m, C**H**=CH_2), 5.03-4.97 (2H, m, CH=C$\mathbf{H_2}$), 4.74-4.71 (1H, m, **H2**), 4.23 (1H, d, *J* 7.2, C**H**OTHP),

3.47-3.28 (2H, m, **H6**), 1.84-1.35 (6H, m, **H3**-**H5**), 0.36 (3H, s, SiC**H$_3$**), 0.33 (3H, s, SiC**H$_3$**); δ_C (75 MHz, $CDCl_3$) 137.2 (**Ph**), 137.0 (**C**H=CH_2), 134.7, 129.6, 128.2 (**Ph**), 113.7 (CH=**C**H$_2$), 95.4 (**C2**), 70.6 (**C6**), 61.1 (**C**HOTHP), 31.0 (**C3**), 26.1 (**C5**), 19.1 (**C4**), -5.0 (Si**C**H$_3$), -5.2 (Si**C**H$_3$). **Fraction 2**: An orange oil, (116 mg, 42%) was isolated as 21:79 mixture of diastereoisomers.

Data: δ_H (300 MHz, $CDCl_3$) 7.60-7.23 (5H, m, **Ph**), 5.91 (1H, ddd, *J* 17.4, 10.8, 6.7, C**H**=CH_2), 5.06 (1H, d, *J* 17.4, CH=C(**H**)H *trans*), 4.94 (1H, d, *J* 10.8, CH=C(**H**)H *cis*), 4.41 (1H, t, *J* 3.1, **H2**), 4.04 (1H, d, *J* 6.7, C**H**OTHP), 3.93-3.85 (1H, m, 1 × **H6**), 3.43-3.37 (1H, m, 1 × **H6**), 1.86-1.36 (6H, m, **H3**-**H5**), 0.32 (3H, s, SiC**H$_3$**), 0.31 (3H, s, SiC**H$_3$**); δ_C (75 MHz, $CDCl_3$) 138.3 (**C**H=CH_2), 134.8, 134.6, 129.6, 128.1 (**Ph**), 111.6 (CH=**C**H$_2$), 100.6 (**C2**), 75.0 (**C6**), 62.9 (**C**HOTHP), 31.0 (**C3**), 26.1 (**C5**), 19.9 (**C4**), -5.0 (Si**C**H$_3$), -5.2 (Si**C**H$_3$). (Combined yield of **15**:208 mg, 75% as a 56:44 mixture of diastereomers). LRMS m/z (ES) 299.1 (100%) $[M+Na]^+$, 277.1 (36) $[M+NH]^+$. HRMS Found $[M+Na]^+$ 299.1440. $C_{16}H_{24}O_2NaSi^{28}$ requires M^+ 299.1443. Found $[M+H]^+$ 277.1617. $C_{16}H_{25}O_2Si^{28}$ requires M^+ 277.1624.

1-(Dimethylphenylsilyl)propan-1-one **17** and ***1-Pent-3-enylpyrrolidine-2,5-dione*** **16**

1-Pent-4-enylpyrrolidine-2,5-dione **5** (35 mg, 0.21 mmol) was dissolved in anhydrous degassed CH_2Cl_2 (1.2 mL, 0.18 M) and was heated to reflux for 30 min. (*R*)-1-(Dimethylphenylsilyl)prop-2-en-1-ol **12** (40 mg, 0.21 mmol) was added followed by the addition of the second generation Grubbs' catalyst tricyclohexylphosphine[1,3-bis(2,4,6-trimethylphenyl)-4,5-dihydroimidazol-2-ylidene][benzylidene]ruthenium (IV) dichloride (18 mg, 0.021 mmol, 5 mol%). The solution became a rose colour and was heated to reflux for 20 h by which time the colour had changed to a golden brown. The reaction vessel was opened to air and stirred for 3 h and the solvent removed *in vacuo*. Purification by column chromatography (petrol:EtOAc, 6:4) yielded two compounds isolated in two fractions. **Fraction 1**: *1-(Dimethylphenylsilyl)propan-1-one* **17** (14 mg, 35%) was isolated as a yellow oil, whose data is ν_{max}/cm^{-1} (Liquid film) 3069 (w, aromatics), 2959 and 2934 (w, aliphatics), 1642 (s, C=O); δ_H (300 MHz, $CDCl_3$) 7.61-7.26 (5H, m, **Ph**), 2.59 (2H, q, *J* 7.2, C**H$_2$**CH$_3$), 0.91 (3H, t, *J* 7.2, CH$_2$C**H$_3$**), 0.49 (6H, s, SiC**H$_3$**); δ_C (75 MHz, $CDCl_3$) 246.1 (**C**=O), 134.6, 140.0, 129.9, 128.2 (**Ph**), 42.0 (**C**H$_2$CH$_3$), 6.1 (CH$_2$**C**H$_3$), -4.7 (Si**C**H$_3$).

Unable to obtain LRMS. Data corresponds to that quoted in the literature. [6] **Fraction 2**: *1-Pent-3-enylpyrrolidine-2,5-dione* **16** (35 mg, 99%) was isolated as a yellow oil. An inseparable mixture of stereoisomers was obtained (*Z:E* 1:3), whose data is ν_{max}/cm^{-1} (Liquid film) 2942 and 2857 (m, aliphatics), 1773 and 1701 (CO-NH-CO).

Data for the major *trans* stereoisomer: δ_H (300 MHz, $CDCl_3$) 5.51-5.31 (2H, m, C**H**=C**H**), 3.53 (2H, t, *J* 7.2, NC**H₂**), 2.69 (4H, s, COC**H₂**C**H₂**CO), 2.25 (2H, q, *J* 7.2, C**H₂**C=C), 1.63 (3H, dd, *J* 6.0, 0.6, C=CC**H₃**); δ_C (75 MHz, $CDCl_3$) 172.6 (**C**=O), 128.5 (**C**H=CHCH₃), 127.1 (CH=**C**HCH₃), 38.9 (N**C**H₂), 31.2 (**C**H₂C=C), 28.5 (CO**C**H₂), 18.3 (C=CH**C**H₃). Signals for the minor *cis* stereoisomer visible at: δ_H (300 MHz, $CDCl_3$) 5.51-5.31 (2H, m, C**H**=C**H**), 3.55 (2H, t, *J* 7.3, NC**H₂**), 2.70 (4H, s, COC**H₂**C**H₂**CO), 2.35 (2H, q, *J* 7.3, C**H₂**C=C), 1.59 (3H, dd, *J* 6.7, 0.8, C=CC**H₃**); δ_C (75 MHz, $CDCl_3$) 127.5 (**C**H=CHCH₃), 126.0 (CH=**C**HCH₃), 38.6 (N**C**H₂), 31.3 (**C**H₂C=C), 21.5 (CO**C**H₂), 13.1 (C=CH**C**H₃). LRMS m/z (ES) 168.1 (100%) $[M+H]^+$.

References

[1] Tarling CA, Holmes AB, Markwell RE, Pearson ND *J. Chem. Soc., Perkin Trans. 1* 1999: 1695-1701.

[2] Hiemstra H, Sno MHAM, Vijn RJ, Speckamp WN *J. Org. Chem.* 1985, **50**: 4014-4020.

[3] Kim S-H, Kim S-I, Lai S, Cha JK *J. Org. Chem.* 1999, **64**: 6771-6775.

[4] Buynak JD, Strickland JB, Lamb GW, Khasnis D, Modi S, Williams D, Zhang H *J. Org. Chem.* 1991, **56**: 7076-7083.

[5] Panek JS, Cirillo PF *J. Am. Chem. Soc.* 1990, **112**: 4873-4878.

[6] Clark CT, Milgram BC, Scheidt KA *Org. Lett.* 2004, **6**: 3977-3980.
